# Metallothionein crypt-restricted immunopositivity indices (MTCRII) correlate with aberrant crypt foci (ACF) in mouse colon

**DOI:** 10.1038/sj.bjc.6602633

**Published:** 2005-05-31

**Authors:** E T Donnelly, H Bardwell, G A Thomas, E D Williams, M Hoper, P Crowe, W G McCluggage, M Stevenson, D H Phillips, A Hewer, M R Osborne, F C Campbell

**Affiliations:** 1 Departments of Surgery, Centre for Cancer Research and Cell Biology, Queen's University of Belfast, Clinical Sciences Building, Grosvenor Road, Belfast BT12 6BJ, Northern Ireland, UK; 2 Strangeways Research Laboratories, Worts Causeway, Cambridge CB1 8RN, UK; 3 Department of Pathology, Centre for Cancer Research and Cell Biology, Queen's University of Belfast, Belfast BT12 6BJ, Northern Ireland, UK; 4 Department of Epidemiology, Centre for Cancer Research and Cell Biology, Queen's University of Belfast, Belfast BT12 6BJ, Northern Ireland, UK; 5 Section of Molecular Carcinogenesis, Institute of Cancer Research, Brookes Lawley Building, Cotswold Road, Sutton, Surrey SM2 5NG, UK

**Keywords:** stem cell, mutation, mixture

## Abstract

Metallothionein (MT) crypt-restricted immunopositivity indices (MTCRII) are colonic crypt stem cell mutation markers that may be induced early and in abundance after mutagen treatment. Metallothionein is the endogenous reporter gene for MTCRII, but is not typically implicated in the classical pathway of colorectal tumorigenesis. Hence, the oncological relevance of MTCRII is unclear. This study tests the hypothesis that MTCRII induced by *N*-methyl-*N*-nitrosourea (MNU) and lambda carrageenan (*λ*CgN) associate with aberrant crypt foci (ACF) in mouse colon. Undegraded *λ*CgN and MNU were tested alone and in combination against MTCRII and ACF in Balb/c mice, at 20 weeks after the start of treatment. MTCRII were unaffected by *λ*CgN alone. Combined *λ*CgN/MNU treatments induced greater MTCRII (*P*<0.01) as well as greater number (*P*<0.001) and crypt multiplicity (*P*<0.01) of ACF than MNU alone. MTCRII were approximately 10-fold more numerous than ACF, although linear correlations were observed between these parameters (*r*=0.732; *P*<0.01). MTCRII are induced by *λ*CgN/MNU interactions in sufficient numbers to provide statistical power from relatively small sample sizes and correlate with ACF formation. MTCRII could thus provide the basis for a novel medium-term murine bioassay relevant to early-stage colorectal tumorigenesis.

Humans are exposed to mixtures of genotoxic and nongenotoxic environmental chemicals that may be linked to cancer (Burkart and Jung, 1998; Minamoto *et al*, 1999). Robust biomarkers of somatic stem cell mutation and mutant clonal expansion may provide cancer surrogates that are useful for risk assessment. Acquired mutation of a selectable endogenous reporter gene like glucose-6-phosphate dehydrogenase (G6PD) within a colonic crypt stem cell induces a crypt-restricted phenotype change (Griffiths *et al*, 1988; Park *et al*, 1995; Kuraguchi *et al*, 1997). Stable, crypt-restricted immunopositivity for metallothionein (MT) is a more recently described stem cell mutation marker for mouse colon that can be assayed in paraffin-fixed tissue sections and has been validated against the G6PD assay (Cook *et al*, 2000). MT-immunopositive crypt frequency has shown a dose response to three different chemical mutagens (Jasani *et al*, 1998; Cook *et al*, 2000; Donnelly *et al*, 2004), which strengthens the evidence that it is a somatic mutation marker.

We have previously shown that a model genotoxic/nongenotoxic chemical mixture, comprising *N*-methyl-*N*-nitrosourea (MNU) and undegraded lambda carrageenan (*λ*CgN), have interactive effects upon MT crypt-restricted immunopositivity indices (MTCRII), including frequency and size of MT-immunopositive foci and total number of MT-immunopositive crypts (Donnelly *et al*, 2004). However, the role of MT mutation in the classical molecular pathway of colorectal cancer is uncertain and thus the oncological relevance of MTCRII is unclear.

This study assesses MTCRII induced by similar MNU/*λ*CgN regimens as in our previous investigation (Donnelly *et al*, 2004) against aberrant crypt foci (ACF) in mouse colon. MTCRII develop within 4–6 weeks of mutagen exposure (Jasani *et al*, 1998; Cook *et al*, 2000). ACF represent the earliest morphological stage of colorectal tumorigenesis (McLellan *et al*, 1991). Some early ACF may regress (Shpitz *et al*, 1996), although large ACF that develop at late intervals after mutagen treatment may have greater risk of dysplasia and cancer (Papanikolaou *et al*, 2000). In this study therefore, assays were conducted at 20 weeks after mutagen treatment to enable assessment of MTCRII against large or persistent ACF.

## MATERIALS AND METHODS

Chemicals, reagents, animals and treatment regimens were as described previously (Donnelly *et al*, 2004). Anti-MT primary antibody (mouse anti-horse monoclonal E9, isotype : IgG1) and peroxidase-conjugated rabbit anti-mouse immunoglobulins were obtained from DAKO Ltd, Ely, Cambridgeshire, UK (Dako M0639 and P0161 respectively), as described previously (Jasani *et al*, 1998; Cook *et al*, 2000). Methylene blue was obtained from BDH Chemicals Ltd, Poole, Dorset, UK (BDH 34048).

### Animals and treatment regimes

Female adult Balb/c mice, aged 6–8 weeks, were obtained from Harlan UK Ltd, Bicester, Oxon, UK, divided into groups of five or 10, ear-punched and placed in coded stainless-steel wire cages, maintained and fed as outlined previously (Donnelly *et al*, 2004). Individual animal weights and group fluid and AIN-76 diet consumption were assessed daily, during weekdays.

### Treatment groups

In all, 90 female adult Balb/c mice aged 6–8 weeks were divided into 11 groups of five or 10 that received no MNU, MNU (62.5 mg kg^−1^ dissolved in dimethylsulphoxide (DMSO)) alone or in combination with 1 or 4% *λ*CgN as follows:

Group 1 (*n*=5): Drinking water only for 20 weeks (water only control).
Group 2 (*n*=5): Single intraperitoneal (i.p.) injection of vehicle (DMSO), then drinking water for 20 weeks (vehicle control).
Group 3 (*n*=5): Continuous 1% *λ*CgN only for 20 weeks.
Group 4 (*n*=5): Continuous 4% *λ*CgN only for 20 weeks.
Group 5 (*n*=10): MNU 62.5 mg kg^−1^ i.p., then drinking water only for 20 weeks.
Group 6 (*n*=10): MNU 62.5 mg kg^−1^ i.p. and 1% *λ*CgN for 7 days during week 1, then drinking water until 20 weeks.
Group 7 (*n*=10): MNU 62.5 mg kg^−1^ i.p. and 4% *λ*CgN for 7 days during week 1, then drinking water until 20 weeks.
Group 8 (*n*=10): MNU 62.5 mg kg^−1^ i.p. and three 7-day treatments of 1% *λ*CgN during weeks 1, 4 and 7. Drinking water was given between and after *λ*CgN treatments until 20 weeks.
Group 9 (*n*=10): MNU 62.5 mg kg^−1^ i.p. and three 7-day treatments of 4% *λ*CgN during weeks 1, 4 and 7. Drinking water was given between and after *λ*CgN treatments until 20 weeks.
Group 10 (*n*=10): MNU 62.5 mg kg^−1^ i.p. and continuous 1% *λ*CgN treatment until 20 weeks.
Group 11 (*n*=10): MNU 62.5 mg kg^−1^ i.p. and continuous 4% *λ*CgN treatment until 20 weeks.

In combined regimens, MNU was administered after the first 5 days of *λ*CgN treatment.

### Welfare considerations and weight index

Animal welfare considerations were strictly in accordance with OECD guidelines (OECD, 2002). Animals were weighed daily and weight index was calculated as the ratio at study completion relative to weight at study start. Values for mean weight index were compared between treatment groups, at study completion.

### Assay of ACF

All assays of ACF were blinded to treatment and carried out after colonic retrieval at 20 weeks after the initiation of treatment. Colons were carefully pinned flat on a cork mat, painted with 0.1% methylene blue and left at room temperature for 10 min. Assay of ACF was performed using a dissecting microscope at × 40 magnification and the following parameters were recorded:

ACF number: Assessed as the total number of ACF per colon or per 10^4^ colonic crypts.
ACF size (crypt multiplicity): Crypt multiplicity was determined as the number of aberrant crypts per ACF.

Colons were then ‘Swiss-rolled’ on the cork mat, with the ileocaecal junction at the centre of the roll, fixed in neutral formal saline for 48 h, and embedded in paraffin wax blocks.

### Assay of MTCRII

All assays of MTCRII were blinded to treatment and carried out 20 weeks after the initiation of treatment. MTCRII were assayed as described previously (Donnelly *et al*, 2004). Briefly, paraffin-embedded sections (4 *μ*m thickness) were cut at 10 levels (L1–L10), 100 *μ*m apart through the ‘Swiss-rolled’ colon. One section from each level was stained using a standard indirect immunoperoxidase technique for MT, while endogenous peroxidase activity was blocked using 3% hydrogen peroxide in methanol. Slides were incubated with an anti-MT primary antibody (E9, isotype : IgG1; 100 *μ*l per slide) (DAKO Ltd, Ely, Cambridgeshire, UK). The secondary antibody used was horseradish peroxidase-conjugated rabbit anti-mouse immunoglobulin (Dako catalogue no. P0161). Negative control sections were incubated either in the absence of antibody, in normal mouse serum (1 : 1000), or with an irrelevant antibody of the same IgG subclass (1 : 1000) (IgG1; Dako catalogue product code X0931). These were consistently negative. Positive control sections included mouse colon previously treated by *N*-ethyl-*N*-nitrosourea (ENU; 250 mg kg^−1^), which induces MT-immunopositive crypts (Cook *et al*, 2000). Sections were washed and 100 *μ*l of the chromogen 3,3′-diaminobenzidine tetrahydrochloride was added before counter-staining with Harris haematoxylin. The frequency and size of MT-immunopositive foci as well as total number of MT-immunopositive crypts were assessed as follows:

MT-immunopositive single crypts or patches of ⩾2 contiguous MT-immunopositive crypts were recognised by their dark brown stain against a haematoxylin background and were assessed in transverse or longitudinal section through the crypt lumen. Each single or contiguous patch of ⩾2 MT-immunopositive crypts was considered to represent a single mutant focus.
The size of each MT-immunopositive patch was assessed by the number of contiguous MT-immunopositive crypts within the patch. Patches were recorded as doubles, triples or greater (*n*=2, 3, etc., MT-immunopositive crypts).
The total number of MT-immunopositive crypts per mouse colon was determined by the sum of single immunopositives and all immunopositives within patches.

Endpoints of the (i) frequency of MT-immunopositive foci (ii) number of MT-immunopositive patches and (iii) the total number of MT-immunopositive crypts were expressed as the number per 10^4^ total crypts, in mouse colon.

### Data analysis

Serial weight data were available in individual mice. The weight index was calculated as the weight at study completion relative to weight at study start, expressed as a percentage. Between-group differences of weight index were assessed by one-way ANOVA. Descriptive statistics applied to weight index were expressed as mean±standard deviation (mean±s.d.). Group data were available for consumption of food and fluid, which were assessed in grams or ml per kg body weight, respectively. Descriptive statistics were expressed as mean±s.d. To achieve a normal distribution, MTCRII and ACF data from each treatment group were log transformed to ensure a normal distribution and assessed by a probability plot of residuals. Transformed data were analysed by univariate ANOVA. Duncan *post hoc* tests were applied to assess differences between specific treatment regimens. Differences of MT-immunopositive patch formation between MNU alone and all combinations of *λ*CgN/MNU were assessed by Student's *t*-test. Correlations between MTCRII and ACF data were investigated by Pearson's product moment coefficient. SPSS for Windows (version 11) was used for statistical analysis (SPSS Inc., Chicago, Il, USA).

## RESULTS

### Food, fluid intake and body weight

In all, 11 treatment groups of mice (*n*=90 total) received water- or vehicle-only controls, high- or low-dose *λ*CgN alone or in combination with MNU (62.5 mg kg^−1^ i.p.). *λ*CgN was given in single or recurrent short- or long-term patterns of exposure. Group values for fluid, food intake and weight index are shown in Table 1. No significant between-group differences of weight index were observed at study completion (Table 1).

### Treatment effects upon MTCRII

**Figure 1:**
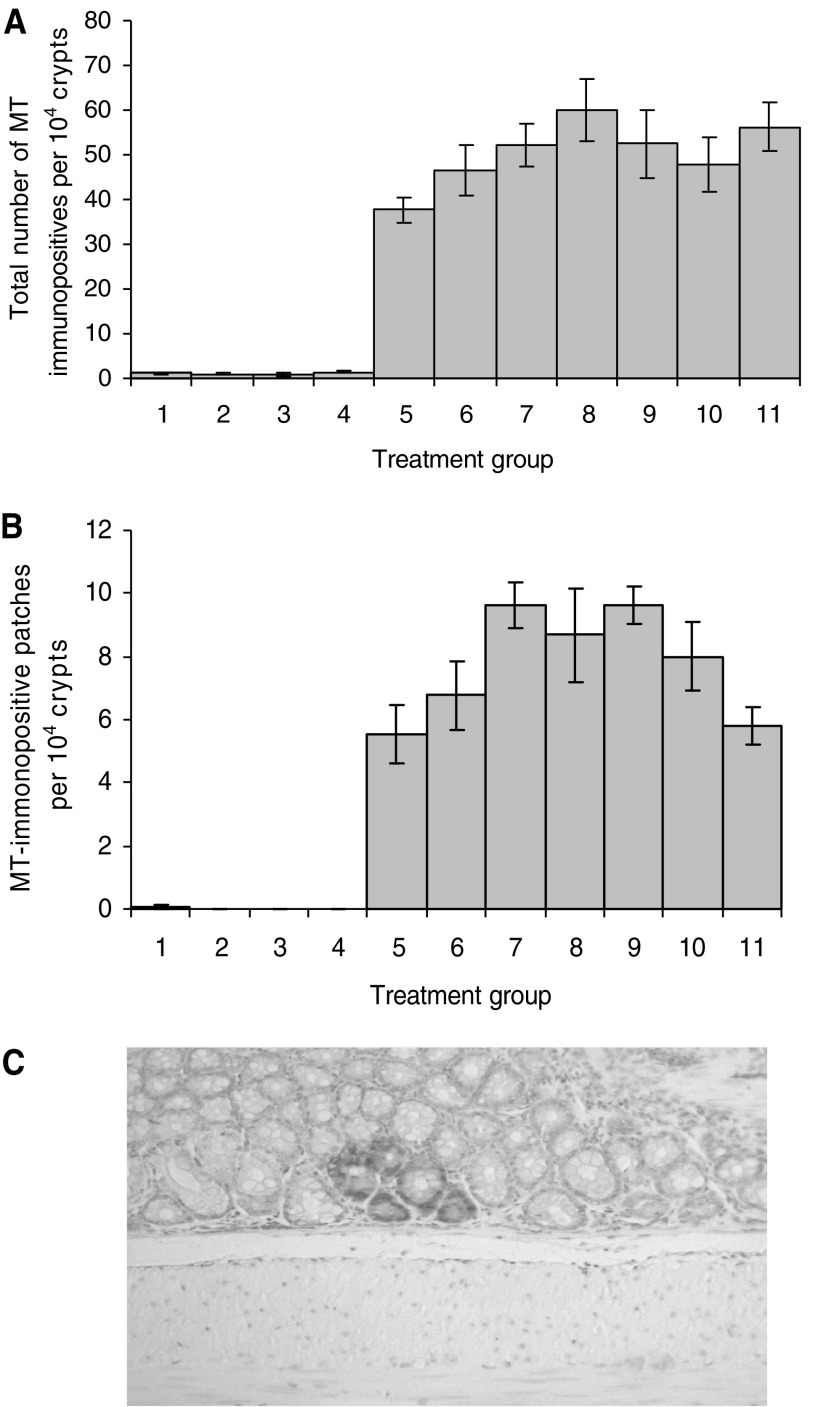
Treatment effects upon MTCRII. (**A**) Total MT-immunopositive crypt number per 10^4^ crypts in each treatment group. Significant between-group treatment differences were observed (*P*<0.01). The Duncan *post hoc* test identified three distinct homogeneous treatment subsets (A–C), showing significant incremental differences in total mutation load: (A) Groups 1–4, distilled water, DMSO or *λ*CgN only (1 and 4%); (B) Groups 5 and 6, MNU only or MNU and one 7 day cycle of 1% *λ*CgN; (C) Groups 8 and 11, MNU and three 7 day cycles of 1% *λ*CgN or MNU and continuous 4% *λ*CgN. Groups 7, 9 and 10 overlapped subsets B and C. (**B**) Formation of patches of ⩾2 contiguous MT immunopositive crypts in each treatment group. The Duncan posthoc test identified three distinct homogeneous treatment subsets (A–C), showing significant incremental differences in mutant (MT immunopositive) patch formation: (A) Groups 1–4, Distilled water, DMSO or *λ*CgN only (1 and 4%); (B) Group 5, MNU alone; (C) Groups 7 and 9, MNU and one or three 7 day cycles of 4% *λ*CgN. Groups 6, 8, 10 and 11 overlapped subsets B and C. (**C**) Large MT immunopositive patch. An example of a large patch comprising 5 MT immunopositive crypts, from a combined *λ*CgN/MNU treatment group.

Group values for MTCRII, including total number of MT-immunopositive crypts, MT-immunopositive patch formation and frequency of MT-immunopositive foci, are shown in Table 2. The total number of MT-immunopositive crypts was increased by >25-fold in excess of that of vehicle alone, by MNU (62.5 mg kg^−1^) treatment, but was unaffected by *λ*CgN treatment alone. Data analysis by one-way between-group ANOVA with the Duncan *post hoc* test allowed division of results into statistically different subsets. Combined *λ*CgN/MNU regimens induced significantly greater total number of MT-immunopositive crypts compared to MNU alone or treatments lacking MNU (*P*<0.01; Table 2). Significant incremental differences were observed between treatment subsets (A–C), where A represents treatment groups 1–4, B represents groups 5 and 6 and C represents groups 8 and 11. Treatment groups 7, 9 and 10 overlapped subsets B and C (Figure 1A).

Significant between-group differences in the frequency of patches of ⩾2 contiguous MT-immunopositive crypts were also observed (*P*<0.05; ANOVA). Significant incremental differences were observed between three treatment subsets (A–C), where A represents treatment groups 1–4, B represents group 5 and C represents groups 7 and 9. Treatment groups 6, 8, 10 and 11 overlapped subsets B and C (Figure 1B). Over 95% of mutant patches involved only two contiguous mutant crypts. The frequency of large MT-immunopositive patches (⩾3 contiguous immunopositive crypts) was 0.38±0.5 per 10^4^ total crypts for MNU alone (group 5) *vs* 1.12±1.01 per 10^4^ total crypts for all MNU/*λ*CgN treatment groups (*P*=0.002). All patches of ⩾4 MT-immunopositive crypts were observed in combined *λ*CgN/MNU treatment groups.

### Effect of treatment regimes on ACF frequency

**Figure 2:**
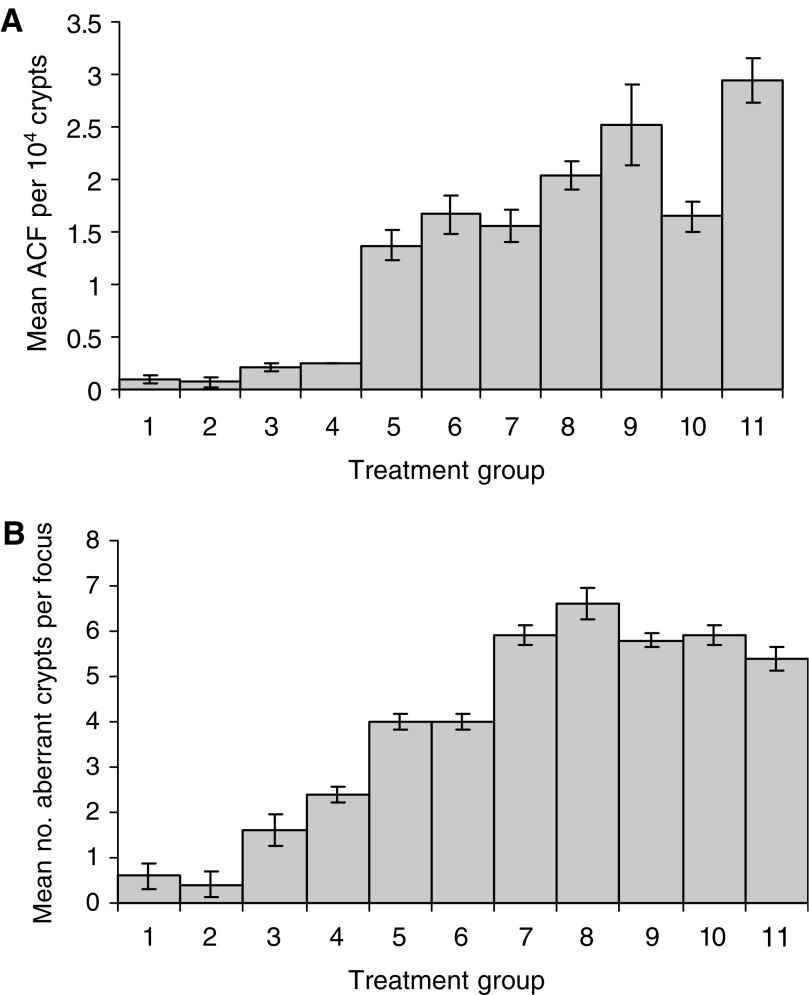
Treatment effects on ACF. (**A**) Effects of treatment regimens on ACF number in murine colon. One-way analysis of variance demonstrated significant between-group differences in numbers of ACF per 10^4^ colonic crypts (*P*<0.001). The Duncan post hoc test identified 5 homogeneous treatment subsets (A–E), showing significant incremental differences in frequency of ACF formation: (A) Groups 1–4, Distilled water, DMSO or *λ*CgN only (1 and 4%); (B) Group 5, MNU alone; (C) Groups 6 and 7, MNU and one 7 day cycle of 1 or 4% *λ*CgN; (D) Group 8, MNU and three 7-day cycles of 1% *λ*CgN; (E) Groups 9 and 11, MNU and either three 7-day cycles of 4% *λ*CgN or continuous 4% *λ*CgN. These subsets showed significant incremental differences of mean ACF per 10^4^ colonic crypts. Group 10 (MNU and 3 × 7-day cycles of 4% *λ*CgN) overlapped subsets C and D. (**B**) Effects of treatment regimens on size of ACF (crypt multiplicity). One-way analysis of variance demonstrated significant between-group differences in numbers of aberrant crypts per focus {crypt multiplicity} (*P*<0.01). The Duncan post hoc test identified 5 homogeneous treatment subsets (A–E), showing significant incremental differences in crypt multiplicity: (A) groups 1 and 2, distilled water alone or DMSO alone; (B) groups 3 and 4, continuous treatment with 1 or 4% *λ*CgN alone; (C) groups 5 and 6, MNU alone or MNU and 1 × 7-day cycle of 1% *λ*CgN; (D) groups 9 and 11, MNU and either 3 × 7-day cycles of 4% *λ*CgN or continuous 4% *λ*CgN; (E) group 8, MNU and three 7-day cycles of 1% *λ*CgN. Groups 7 and 10 (MNU and 1 × 7-day cycle of 4% *λ*CgN or continuous 1% *λ*CgN) overlapped subsets D and E.

The administration of MNU led to a significant increase in ACF numbers by about 10-fold in excess of that of DMSO vehicle alone. ACF data were expressed either as a number per 10^4^ total colonic crypts (Figure 2A) or per mouse colon (Table 3). *λ*CgN treatment alone led to a small significant increase in ACF size, in terms of crypt multiplicity but had no significant effect on ACF number. Combined *λ*CgN/MNU regimens significantly increased ACF number and size (*P*<0.001; ANOVA). *Post hoc* analysis demonstrated significant incremental differences in ACF number between five homogeneous treatment subsets (A–E), where A represents treatment groups 1–4, B represents treatment group 5, C represents groups 6 and 7, D represents group 8 and E represents groups 9 and 11. Group 10 overlapped subsets C and D (Figure 2A; Table 3). Significant effects of treatment on ACF size were also observed (*P*<0.01; Figure 2B) with incremental differences in crypt multiplicity within ACF between five treatment subsets (A–E), where A represents treatment groups 1 and 2, B represents 3 and 4, C represents group 5 and 6, D groups 9 and 11 and E represents group 8. Groups 7 and 10 overlapped subsets D and E.

### Correlations between MTCRII and ACF

**Figure 3:**
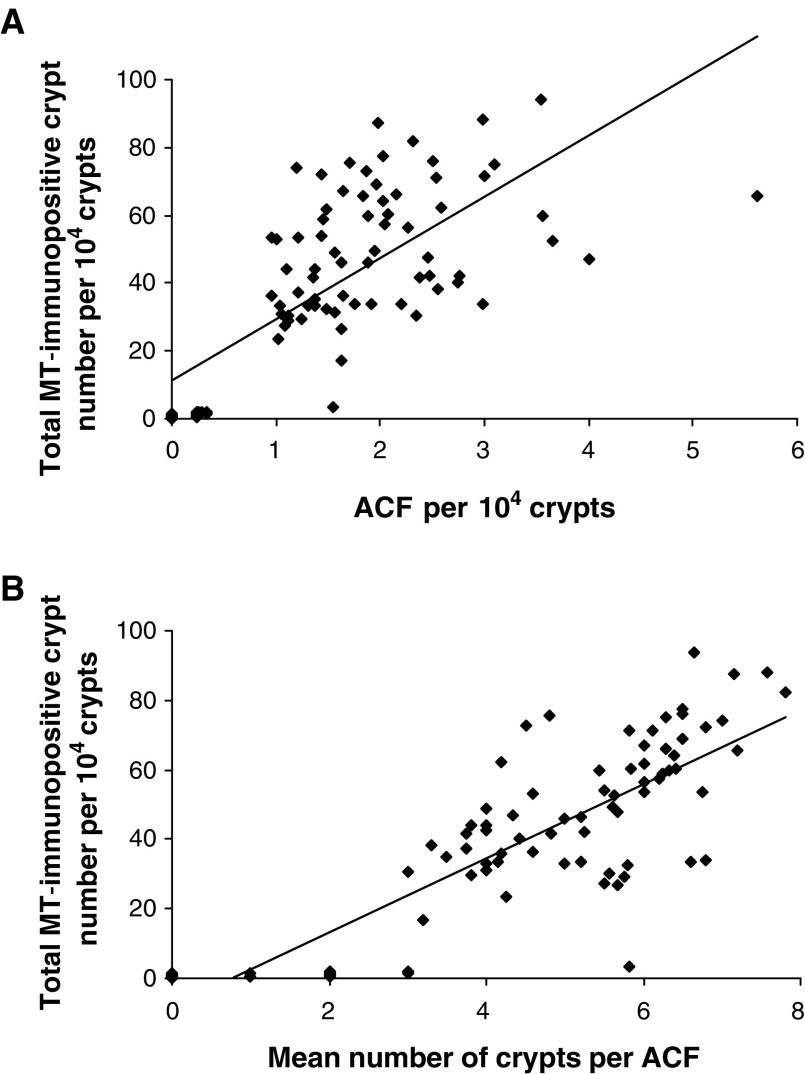
Correlations between MTCRII and ACF. (**A**) Total MT-immunopositive crypt number *vs* number of ACF per 10^4^ crypts. Correlation between total MT-immunopositive crypt number and ACF per 10^4^ crypts in all treatment groups (*r*=0.732; *P*<0.01 by Pearson's product moment test). (**B**) Total MT-immunopositive crypt number *vs* size of ACF. Correlation between total MT-immunopositive crypt number per 10^4^ crypts and ACF size in all treatment groups (*r*=0.84; *P*<0.01 by Pearson's product moment test).

Assessments of MTCRII and ACF were conducted in all treatment groups (1–11). Linear correlations were observed between total MT-immunopositive crypt number and ACF number per 10^4^ crypts (*r*=0.732; *P*<0.01) (Figure 3A) and ACF size, in terms of the number of aberrant crypts per focus (*r*=0.84; *P*< 0.01) (Figure 3B).

## DISCUSSION

Colonic tumorigenesis involves acquisition of mutations or heritable epigenetic events, affecting growth control or differentiation genes within crypt stem cells, progression to premalignant stages including ACF (Bird and Good, 2000) and ultimate invasive carcinoma. Since these events are stochastic, a higher stem cell mutation rate may accelerate distinct stages of this process (Herrero-Jimenez *et al*, 1998). Robust biomarkers of stem cell mutation may thus provide useful surrogates of tumorigenesis. Metallothionein crypt-restricted immunopositivity indices provide a stem cell mutation marker that is initiated by mutagen exposures, yet mimics sporadic tumorigenesis because it occurs in widely scattered single crypts or foci throughout the otherwise normal colon (Cook *et al*, 2000). Since the relationship of MT crypt-restricted immunopositivity to tumorigenesis was unclear, we assessed the relationship between MTCRII and ACF frequency, in mice treated by *λ*CgN and MNU.

The present study uses a similar combinatorial design, involving a single MNU treatment (62.5 mg kg^−1^) together with single, repeated or continuous exposures to low- (1%) or high- (4%) dose *λ*CgN, to that of our previous study (Donnelly *et al*, 2004). In the present study however, follow-up and continuous *λ*CgN treatment were continued for longer term (20 weeks). The present study supports our earlier work and shows that *λ*CgN alone does not significantly affect MTCRII, but enhances MNU effects upon this end point (Donnelly *et al*, 2004). However, sequential or prolonged *λ*CgN exposure to 20 weeks was associated with the development of larger MT-immunopositive (mutant) patches than observed at 10 weeks, in our previous study.

Hence, prolonged *λ*CgN exposure may have cumulative effects upon mutant patch size. These effects could be related to *λ*CgN-induced tissue injury in mouse colon (Donnelly *et al*, 2004), fission of immunopositive crypts and formation or enlargement of immunopositive patches, during continual or repeated regenerative healing.

While biomarkers of rate-limiting steps of tumorigenesis are informative, validation against tumour-associated end points is important. Aberrant crypt foci comprise a contiguous collection of crypts that have thickened epithelia, altered luminal openings and are clearly circumscribed from adjacent normal crypts (Bird, 1987). Gene mutations that are commonly observed in colon cancers including K-ras and APC are also observed in a proportion of ACF (Pretlow *et al*, 1993; Smith *et al*, 1994). Aberrant crypt foci are thus considered to represent early-stage colorectal tumorigenesis (Bird, 1987; Tudek *et al*, 1989; Takayama *et al*, 1998; Bird and Good, 2000), although large or persistent ACF may have greater cancer risk (Papanikolaou *et al*, 2000). The present study has shown that MTCRII may reflect combined effects of chemicals within a mixture, are induced in sufficient numbers to provide statistical power from relatively small animal samples and correlate with ACF formation at 20 weeks after the initiation of treatment. MTCRII may thus provide the basis for an intermediate risk assessment model for diet- or lifestyle-related genotoxic/nongenotoxic chemical combinations, relevant to colonic health.

## Figures and Tables

**Table 1:** Effects of 20-week treatments on group fluid and food consumption and weight index

**Treatment regimes**	**Mice (*n*)**	**Group fluid intake (ml g^−1^ body weight)**	**Group food intake (g g^−1^ body weight)**	**Weight index**
1. Control (water) only	5	0.165	0.184	132.72±2.2
2. DMSO vehicle only	5	0.163	0.192	133.71±2.5
3. Continuous 1% *λ*CgN	5	0.172	0.174	126.51±3.4
4. Continuous 4% *λ*CgN	5	0.113	0.184	132.09±4.7
5. MNU (62.5 mg kg^−1^)	10	0.152	0.161	126.58±6.5
6. MNU+1 × 7 day 1% *λ*CgN	10	0.151	0.170	130.49±6.9
7. MNU+1 × 7 day 4% *λ*CgN	10	0.149	0.165	126.43±5.4
8. MNU+3 × 7 day 1% *λ*CgN	10	0.149	0.167	133.29±6.9
9. MNU+3 × 7 day 4% *λ*CgN	10	0.122	0.165	129.95±3.9
10. MNU+continuous 1% *λ*CgN	10	0.137	0.164	130.22±4.6
11. MNU+continuous 4% *λ*CgN	10	0.114	0.165	125.41±6.2

DMSO=dimethylsulphoxide; *λ*CgN=lambda carrageenan; MNU=*N*-methyl-*N*-nitrosourea.

**Table 2:** Treatment effects upon MTCRII

**Treatment**	**Mice *(n)***	**Total crypt no.**	**Total MT-immunopositive crypt number**	**Patches ⩾2 MT-immunopositive crypts**	**Frequency of MT-immunopositive foci**
1. Water only	5	36 433±2053	1.134±0.34	0.10±0.06	1.130±0.33
2. DMSO only	5	33 292±2888	0.974±0.29	0	0.97±0.29
3. Continuous 1% *λ*CgN	5	37 025±2091	0.88±0.32	0	0.88±0.33
4. Continuous 4% *λ*CgN	5	38 657±1149	1.31±0.32	0	1.31±0.32
5. MNU only (62.5 mg kg^−1^)	10	29 750±1925	37.66±2.93	5.54±0.91	31.73±2.66
6. MNU+1 × 7-day cycle 1% *λ*CgN	10	27 715±1715	46.54±5.63	6.76±1.10	38.60±5.18
7. MNU+1 × 7-day cycle 4% *λ*CgN	10	32 539±1543	52.31±4.82	9.61±0.78	39.39±4.72
8. MNU+3 × 7-day cycles 1% *λ*CgN	10	30 651±2091	59.85±6.91	8.27±1.56	51.46±6.81
9. MNU+3 × 7-day cycles 4% *λ*CgN	10	30 640±3176	52.47±7.80	9.62±0.67	43.79±5.89
10. MNU+continuous 1% *λ*CgN	10	31 372±1832	47.86±5.96	8.00±1.14	38.14±5.11
11. MNU+continuous 4% *λ*CgN	10	23 450±1007	56.23±5.41	5.82±0.69	49.98±5.09

MTCRII=metallothionein crypt-restricted immunopositivity indices; MT=metallothionein; DMSO=dimethylsulphoxide; *λ*CgN=lambda carrageenan; MNU=*N*-methyl-*N*-nitrosourea.

**Table 3:** Treatment effects upon number and size of ACF

**Group**	**Treatment**	**Mice (*n*)**	**No. of ACF per colon**	**No. of ACF per 10^4^ crypts**	**No. of aberrant crypts per ACF**
1	Water only	5	0.40±0.24	0.09±0.04	0.60±0.28
2	DMSO only	5	0.20±0.20	0.07±0.04	0.40±0.25
3	Continuous 1% *λ*CgN	5	0.80±0.20	0.21±0.04	1.60±0.34
4	Continuous 4% *λ*CgN	5	1.00±0	0.25±0.005	2.40±0.24
5	MNU only	10	3.90±0.23	1.37±0.015	4.00±0.17
6	MNU+1 × 7-day cycle 1% *λ*CgN	10	5.00±0.29	1.67±0.18	4.00±0.16
7	MNU+1 × 7-day cycle 4% *λ*CgN	10	4.90±0.23	1.56±0.14	5.90±0.21
8	MNU+3 × 7-day cycle 1% *λ*CgN	10	5.80±0.24	2.04±0.14	6.58±0.33
9	MNU+3 × 7-day cycle 4% *λ*CgN	10	6.70±0.21	2.52±0.39	5.80±0.17
10	MNU+continuous 1% *λ*CgN	10	5.00±0.25	1.65±0.14	5.90±0.22
11	MNU+continuous 4% *λ*CgN	10	6.80±0.29	2.95±0.21	5.38±0.26

ACF=aberrant crypt foci; DMSO=dimethylsulphoxide; *λ*CgN= lambda carrageenan; MNU=*N*-methyl-*N*-nitrosourea.
